# Combined Intracapsular And Extracapsular Neck Of Femur Fractures Case Series, Literature Review And Management Recommendations

**DOI:** 10.2174/1874325001711010600

**Published:** 2017-07-31

**Authors:** Wasim Khan, Rhodri Williams, Sam Hopwood, Sanjeev Agarwal

**Affiliations:** Cardiff & Vale Orthopaedic Centre, Llandough University Hospital, Cardiff & Vale NHS Trust, Cardiff, UK

**Keywords:** Segmental neck of femur fractures, Combined neck of femur fracture, Total hip replacement, Hemiarthroplasty, Constraint, Internal fixation

## Abstract

Concomitant ipsilateral intracapsular and extracapsular fractures of the femoral neck are rare injuries with only 14 cases described in the literature as single case reports. We present three cases that were successfully and uniquely treated by uncemented hip arthroplasties. Two patients underwent complex primary uncemented total hip replacements, and one patient underwent an uncemented bipolar fluted stem hemiarthroplasty. The level of bearing constraint varied between implants. After describing our cases we review the literature and make recommendations on the management of these injuries. We believe that these are significant injuries and best functional results can be achieved with an early diagnosis and patient-specific approach that can include a total hip replacement in appropriate cases.

## INTRODUCTION

Concomitant ipsilateral intracapsular and extracapsular fractures of the femoral neck, otherwise known as segmental neck of femur fractures, are rare injuries but are difficult to manage. These are generally associated with either significant trauma in young patients or low energy injuries to pathological bone in older patients. These injuries are associated with a significant risk of complications including avascular necrosis, non-union and malunion, potentially greater than those associated with single fractures. There has been a limited number of single case reports described in the literature where these fractures are managed with internal fixation or hemiarthroplasty.

## CASE SERIES

We present three cases with segmental neck of femur fractures successfully managed with total hip replacements and a hemiarthroplasty. In our series, two patients received complex primary uncemented total hip replacements and the third patient received a Wagner modular, taper-fluted titanium stem with a bipolar head (Zimmer). One total hip replacement included a constrained hip liner system. This is the first report of the management of these fractures with total hip replacements. Following a description of our cases we review the literature and make recommendations on the management of these challenging fractures.

**Case 1:** A 66 year old male sustained a low energy fall. He was a residential home resident with a history of previous alcoholism and cognitive impairment. Although he resided in a home, prior to the fall he enjoyed a degree of independence and regularly walked to the shops. The patient on radiographs had a displaced intracapsular and intertrochanteric fracture (Fig. **1a**). The patient had a high risk of fixation failure in view of his age, associated risk factors, and fracture configuration. In view of this, a decision was made to perform an arthroplasty. The complicating factors were the patient’s cognitive impairment and abductor insufficiency secondary to the trochanteric fracture. To address these factors, the patient underwent a complex primary total hip replacement with a constrained liner and trochanteric grip plate (Fig. **1b**). At final follow-up at 18 months he was pleased with the results of surgery and his radiographs were satisfactory. There were no recorded complications. He was mobilising unaided and still managing to go to the shops.

**Case 2:** An 82 year old independent male with a history of hip osteoarthritis had a simple mechanical fall sustaining an intracapsular fracture with concominant subtrochanteric fracture (Fig. **2a**). Following radiographs, a computerized tomography (CT) scan was performed to better define the fracture configuration and demonstrated fracture comminution. Due to the segmental nature of the fracture and the pre-existing severe arthritis, fixation was not considered a valid option, and the patient underwent a total hip replacement with plate stabilisation for the fracture extension (Fig. **2b**). There were no recorded complications. At final follow-up 12 months post-operatively he was pain free mobilizing with a walking stick and had satisfactory radiographs.

**Case 3:** Our third case was an 80 year old nursing home resident with multiple co-morbidities who mobilized with a Zimmer frame. She had a fall and radiographs revealed an intracapsular fracture with concominant subtrochanteric fracture with diaphyseal extension (Fig. **3a**). She had an American Society of Anaesthesiologists (ASA) grade of 3. Various surgical treatments were considered, and to reduce the chances of any revision surgery from failure of fixation, an arthroplasty was performed. A primary femoral stem was not appropriate in view of the fracture configuration and extension, therefore an uncemented modular, taper-fluted titanium stem with a bipolar head was used (Fig. **3b**). There were no recorded complications. At final follow-up at two years post-operatively, the patient remained mobile with a Zimmer frame.

## LITERATURE REVIEW

**Fig. (4):**
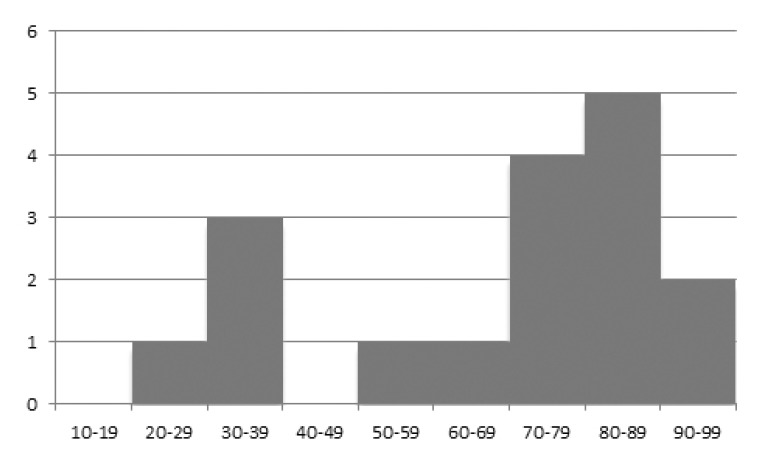
Graph demonstrating the age ranges on the x-axis and the number of cases described inth eliterature on the y-axis. A bimodal distribution is demonstrated.

A review of the literature was performed and 14 cases reports describing 14 segmental neck of femur fractures were identified ranging from 1989 to 2014 [1-14]. The details of these cases are described in (Table **1**). The age ranges of the 14 patients described in the literature and our three patients were plot against the numbers (Fig. **4**) to demonstrate a bimodal distribution of these injuries, similar to other fractures of the neck of femur. The mean age of all patients was 68 years (range 28-97 years). All four patients under the age of 50 years sustained their injuries following a road traffic accident, and 10 of the 12 patients over the age of 60 years had a low energy fracture. The fracture configurations varied, and in five patients additional imaging was performed in addition to radiographs.

In four of the 14 cases described in the literature, the fractures were not initially appreciated, and in two cases further imaging was performed to investigate the fracture configuration further. An *et al.* [1] appreciated the additional fracture when the patient had repeat radiographs whilst waiting to be medically optimised for surgery. Cohen & Rzetelny [3] noticed the additional fracture on intra-operative fluoroscopic screening. Perry & Scott [11] only noticed the intracapsular fracture once it displaced after 10 weeks of mobilisation following dynamic hip screw fixation of the intertrochanteric fracture. Neogi *et al.* [13] only identified the extent of the fracture on CT scanning of the contralateral fracture dislocated hip. Three of the six patients with high energy injuries had significant associated injuries. Interestingly three of seven low energy injuries described in the literature were noticed to have arrhythmias on presentation that needed to be managed before surgery.

The 14 cases reported in the literature so far have been managed in a heterogeneous fashion. Eleven cases underwent fixation with dynamic hip screws, dynamic condylar screws or similar constructs. Three patients underwent hemiarthroplasties. The 14 cases previously described in the literature were followed up for on average of 15 months (range 1-58 month). Interestingly, most patients did well with only one report of avascular necrosis (AVN) [5]. The patient whose fixation failed as the extent of fracture was not recognised intraoperatively refused further surgery [11]. One patient with a high energy injury ended up with a 2cm shortening [7]. One patient died shortly after surgery from other causes [4].

## DISCUSSION

Older patients with low energy fractures need to be optimised before surgery and this may need input from medical and anaesthetic teams [15]. There are a number of surgical treatment options available for neck of femur fractures [16]. The AVN rate of intracapsular fractures depends on the age of the patient; the rate is 20% in patients younger than 60 years old, and 12.5% in patients between the ages of 60 and 80 years old [17]. The rate is likely to be higher in patients with segmental injuries due to the extent of bony and soft tissue disruption. This needs to be borne in mind when considering the optimal surgical management. Although our patients did well following arthroplasty, the literature, albeit with short follow-ups, does suggest good results with internal fixation. Cement augmentation of internal fixation has been described and may further reduce the incidence of complications in these difficult injuries [18]. There is increasing evidence that elderly patients with displaced neck of femur fractures do better with arthroplasty than with internal fixation [19].

We believe that arthroplasty alleviates the risk of AVN, non-union and mal-union associated with fracture fixation and pathological bone, and also allows a more constrained implant where there are concerns regarding stability. In our case series, the level of bearing constraint varied between cases, and this too is an important consideration in deciding the arthroplasty implants. We considered a greater level of constraint of a cup in Case 1 as the patient had a history of cognitive impairment and alcoholism, and a hip fracture configuration suggesting abductor deficiency. We advocate the use of uncemented implants, without potential cement interposition at the fracture site to ensure union.

One limitation of our case series is that it is a retrospective series from a single centre. The cases were managed according to the preference of the operating surgeon and hence different implants were used. These nevertheless highlight that arthroplasty is a valid option where the risks of internal fixation are high.

## RECOMMENDATIONS

We recommend a high index of suspicion when assessing radiographs, and further imaging where the radiographs do not demonstrate the fracture pattern clearly. The management of high energy injuries needs to follow appropriate protocol and the presence of distracting injuries should be considered when assessing for injuries. Although the literature suggests that internal fixation is appropriate for healthier and younger patients, there is increasing evidence that elderly patients and those with co-morbidities with displaced neck of femur fractures do better with arthroplasty.

## CONCLUSION

In conclusion, these fractures are rare but present a challenging problem. We believe that these are significant injuries and best functional results can be achieved with an early diagnosis and patient-specific approach that can include a total hip replacement in appropriate cases.

## Figures and Tables

**Fig. (1a):**
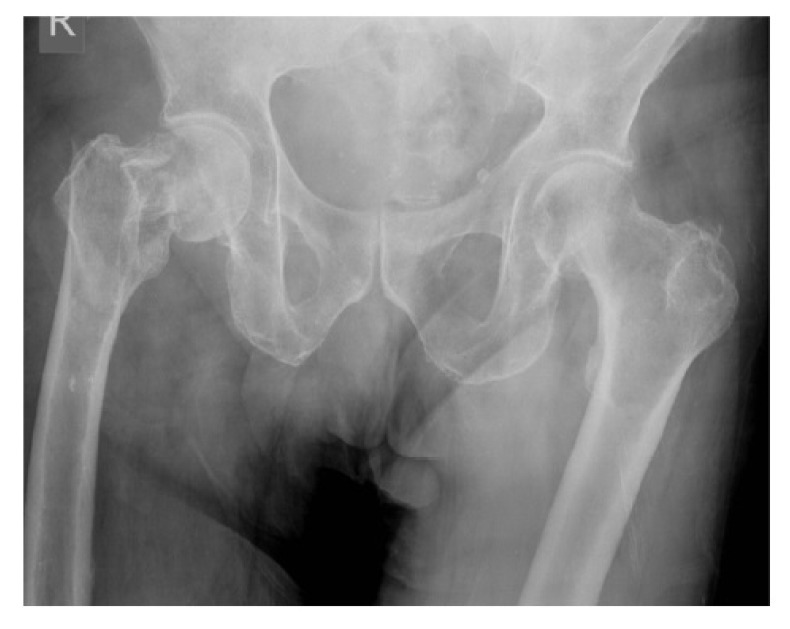
Anterio-posterior pelvic radiograph of Case 1 following the fall (a).

**Fig. (1b):**
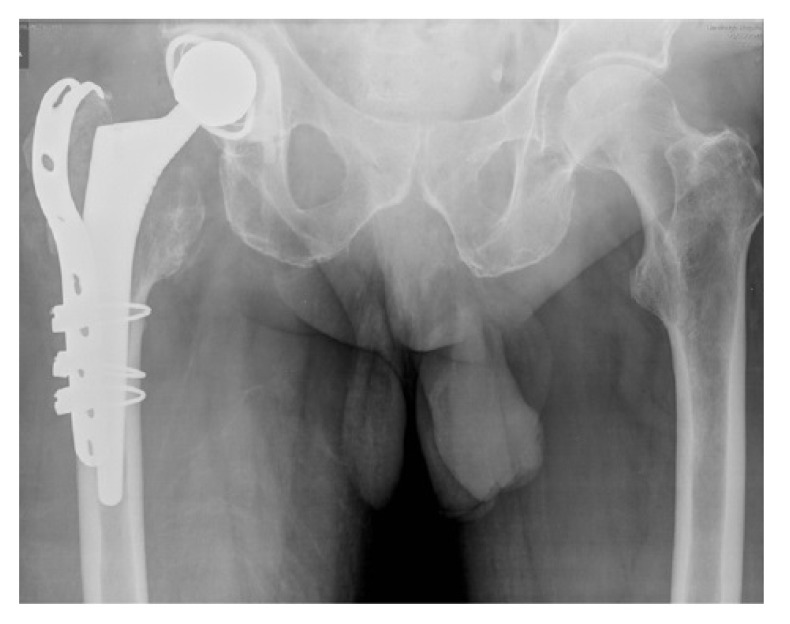
and after total hip replacement and insertion of trochanteric grip plate (b).

**Fig. (2a):**
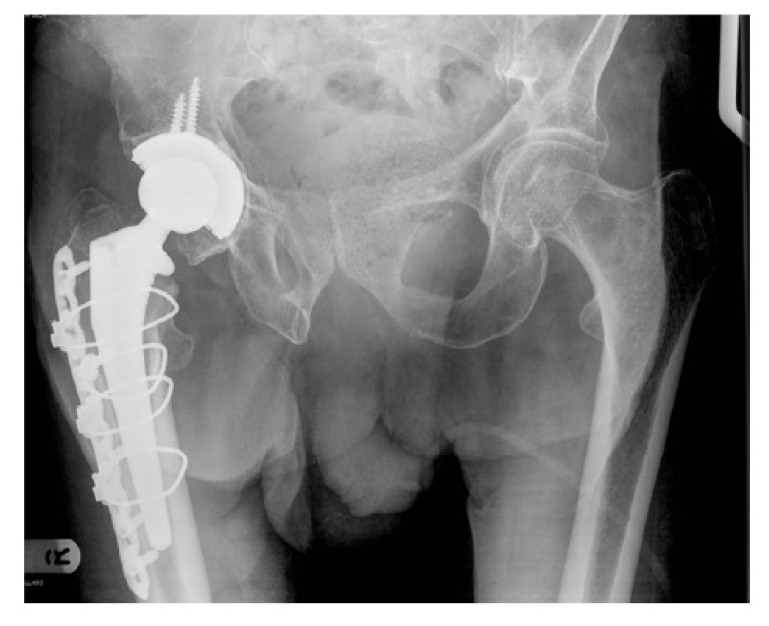
Anterio-posterior pelvic radiograph of Case 2 following the fall (a).

**Fig. (2b):**
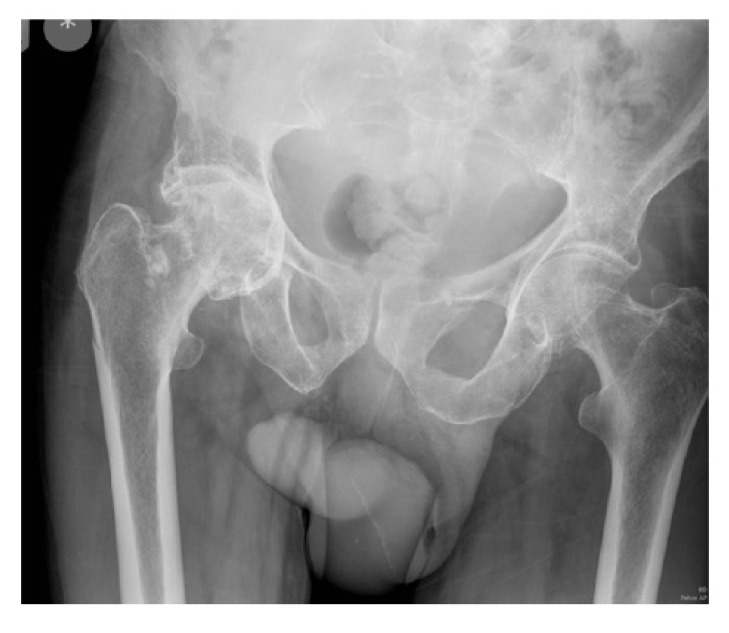
and after total hip replacement with plate stabilisation (b).

**Fig. (3a):**
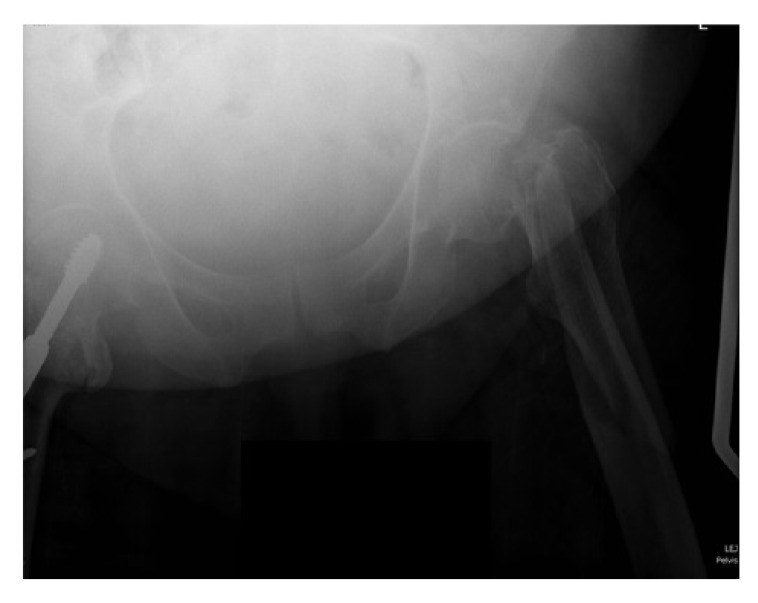
Anterio-posterior pelvic radiograph of Case 3 following the fall (a).

**Fig. (3b):**
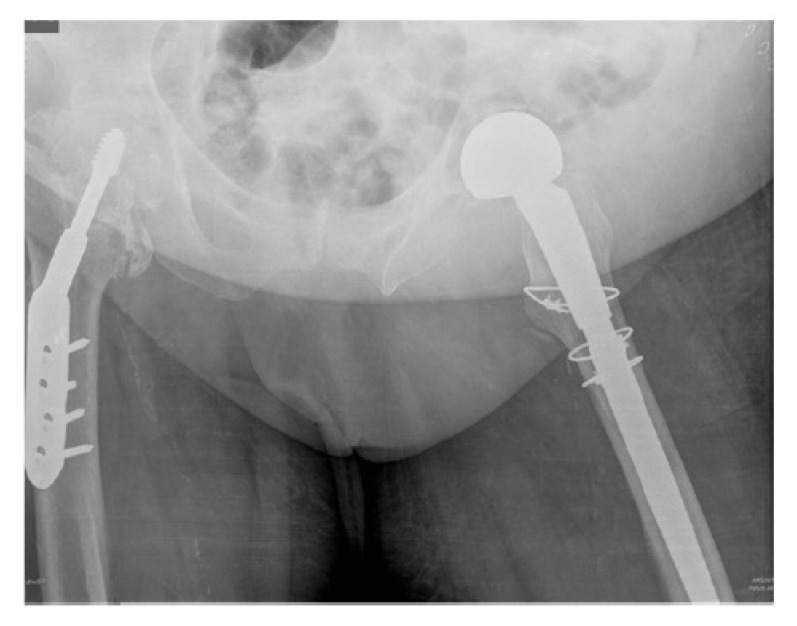
and after bipolar long-stem hemiarthroplasty (b).

**Table 1:** Details of the 14 cases described in the literature.

**Author**	**Sex/ Age**	**Mechanism**	**Fracture Configuration**	**Imaging**	**Difficulty in diagnosis**	**Associated injuries and comorbidities**	**Management**	**Follow up**	**Outcome and complications at final follow-up**
An *et al.* 1989 [1]	97M	Twisting fall	Four-part intertochanteric & subcapital fracture	Radiographs	Subcapital fracture on subsequent imaging whilst patient in traction awaiting medical optimisation	Arrythmia requiring cardioversion preoperatively	Long porous coated stem with a bipolar head hemiarthroplasty and cerclage wires. Bone grafting to medial cortex.	8 months	Asymptomatic
Pemberton *et al.* 1989 [2]	73 F	Fell getting out of bed	Subcapital Garden IV & basal cervical fracture	Radiographs and isotope bone scan	Radioisotope bone scan to confirm acute nature of both fractures	Nil	Five hole DHS	30 months	No problems relating to hip. No evidence of AVN on radiographs or isotope bone scan.
Cohen & Rzetelny 1999 [3]	79 F	Fall at home	Comminuted pertrochanteric & subcapital fracture	Rdiographs	Subcapital fracture noticed intraoperatively on fluoroscopic screening	Nil	Four hole DHS	24 months	Painfree, mobilising with a stick
Lawrence & Isaacs 1993 [4]	72F	Run over by a car	Intertrochanteric & subcapital Garden II fracture	Radiographs and CT	Suspected subcapital fracture requiring CT scan for delineation	Contralateral pubic rami fractures, pulmonary contusions	Four hole DHS	1 month	Satisfactory radiographs. Discharged to hospice at 2 months and died shortly afterwards of metastatic bowel carcinoma
Kumar *et al.* 2001 [5]	83 F	Slid down couch landing directly on hip	Comminuted intertrochanteric & subcapital Garden II fracture	Radiographs	Nil	Arryhthmia requiring correction preoperatively	Derotation screw, five hole DHS, and trochanteric grip plate	12 months	FWB with no hip pain. Radiographs with satisfactory healing and minimal head collapse, Bone scan with evidence of AVN.
Lakshmanan & Peehal 2005 [6]	91 F	Fell from bed	Intracapsular fracture extending to the extracapsular lesser trochanter	Radiographs	Nil	Nil	Cemented hemiarthroplasty	6 moths	Satisfactory clinically and radiographically
Sayegh *et al.* 2005 [7]	54 M	Crush injury in an olive press	Pertrochanteric and subcapital fracture with a nondisplaced greater trochanter	Radiographs	Nil	Extensive soft tissue injury to ipsilateral distal third femur and knee, and closed fracture to ipsilateral humerus	Open reduction and 5 hole DHS and cerclage wire	58 months	2cm shortening clinically, but satisfactory radiographs with union.
Butt *et al.* 2007 [8]	30 M	RTA	Intracapsular and & reverse oblique intertrochanteric fracture	Radiographs	Nil	Nil	DHS with derotation screw	12 months	Pain free with no AVN
Poulter & Ashworth 2007 [9]	76 F	Not stated	Minimally displaced intertrochanteric & slightly angulated subcapital fracture	Radiographs	Nil	Nil	Percutaneous compression plate (two sliding screws in barrels with a plate)	4 months	FWB, no pain, good ROM. Satisfactory radiographs at 3 months.
Dhar *et al.* 2008 [10]	30 M	RTA	Femoral neck and trochanteric reverse oblique fracture	Radiographs	Nil	Nil	Two intertrochanteric lag screws, a DCP, and two cannulated neck screws.	12 months	Pain free with no AVN
Perry & Scott 2008 [11]	86 F	Fall at home	Displaced intertrochanteric & undisplaced intracapsular fracture	Radiographs	Intracapsular fracture missed on initial radiographs and only appreciated once displaced following DHS fixation and 10 weeks of mobilisation	Nil	Four hole DHS	3 months	Fixation failed despite 4 weeks of protected weight bearing, but patient refused further surgery
Loupasis *et al.* 2010 [12]	36 M	Motorcyclist thrown after head on collision with car	Displaced intertrochanteric & subcapital Garden II fracture	Radiographs	Nil	Nil	Three hole DHS with a derotation screw	24 months	Asymptomatic, resumed normal activities. Harris hip score 93.0. Radiographs satisfactory with no AVN.
Neogi *et al.* 2011 [13]	28 M	Front seat unrestrained passenger involved in RTA	Reverse oblique trochanteric and minimally displaced intracapsular fracture	Radiographs and CT	Intracapsular fracture only identified on CT scans performed for contralateral hip investigations	Contralateral posterior hip dislocation, posterior acetabular fracture and femoral shaft fracture.	DCS and derotation screw	28 months	Good fuctional outcome with no AVN
Tahir *et al.* 2014 [14]	87 F	Fall at nursing home	Minimally displaced intertrochanteric and subcapital fracture	Radiographs and CT	Nil	Cardiac arrhythmias noticed on admission	Cemented bipolar hemiarthroplasty and trochanteric plate	3 months	Postoperative wound discharge requiring vaccum dressing. At final follow-up, improving mobility and satisfactory radiographs.

AVN= Avascular Necrosis, RTA= Road Traffic Accident, CT= Computerised Tomography, DHS= Dynamic Hip Screw, FWB= Full Weight Bearing, DCS= Dynamic Condylar Screw, ROM= Range of Movement.
